# An machine learning model to predict quality of life subtypes of disabled stroke survivors

**DOI:** 10.1002/acn3.51960

**Published:** 2023-12-07

**Authors:** Qi Xu, Lei Lei, Zhenguo Lin, Weimin Zhong, Xinhong Wu, Dingzhao Zheng, Taibiao Li, Jiyi Huang, Tiebin Yan

**Affiliations:** ^1^ Xiamen Fifth Hospital Xiamen 361101 China; ^2^ Department of Clinical Medicine Xiamen Medical College Xiamen 361023 China; ^3^ Department of Clinical Medicine The First Affiliated Hospital of Xiamen University Xiamen 361003 China; ^4^ Department of Rehabilitation Medicine Sun Yat‐sen Memorial Hospital, Sun Yat‐sen University Guangzhou 510120 China; ^5^ The Engineering Technology Research Center of Rehabilitation and Elderly Care of Guangdong Province Guangzhou 510120 China

## Abstract

**Objective:**

Stroke causes serious physical disability with impaired quality of life (QoL) and heavy burden on health. The goal of this study is to explore the impaired QoL typologies and their predicting factors in physically disabled stroke survivors with machine learning approach.

**Methods:**

Non‐negative matrix factorization (NMF) was applied to clustering 308 physically disabled stroke survivors in rural China based on their responses on the short form 36 (SF‐36) assessment of quality of life. Principal component analysis (PCA) was conducted to differentiate the subtypes, and the Boruta algorithm was used to identify the variables relevant to the categorization of two subtypes. A gradient boosting machine(GBM) and local interpretable model‐agnostic explanation (LIME) algorithms were used to apply to interpret the variables that drove subtype predictions.

**Results:**

Two distinct subtypes emerged, characterized by short form 36 (SF‐36) domains. The feature difference between worsen QoL subtype and better QoL subtype was as follows: role‐emotion (RE), body pain (BP) and general health (GH), but not physical function (PF); the most relevant predictors of worsen QoL subtypes were help from others, followed by opportunities for community activity and rehabilitation needs, rather than disability severity or duration since stroke.

**Interpretation:**

The results suggest that the rehabilitation programs should be tailored toward their QoL clustering feature; body pain and emotional‐behavioral problems are more crucial than motor deficit; stroke survivors with worsen QoL subtype are most in need of social support, return to community, and rehabilitation.

## Introduction

Around the world, there are 12.2 million new strokes per year (one every 3 sec). About 101 million people are living poststroke, a number that has nearly doubled in the last 30 years.^1^ Many stroke survivors experience long‐term dysfunction, especially physical disability.^2^ Globally, stroke is the third leading cause of death and disability combined (expressed in terms of disability‐adjusted life‐years lost (DALYs)). In addition, the cost burden of stroke is rising. The global cost of stroke in 2017 was estimated at US $861 billion, or about 1.12% of global GDP. Low‐ and middle‐income countries account for 89% of global stroke deaths and disability.^1^ China has one of the highest burdens, especially in its rural areas.^3^ That makes finding clustered clinical features among physically disabled stroke survivors in rural China important. Defining them will help society develop more precise prevention and rehabilitation measures.

Beyond quality of life (QoL), clinical features commonly used to assess rehabilitation outcomes after stroke commonly include functional assessment, and muscle performance.^4^ QoL has been a key indicator for evaluating the effectiveness of stroke rehabilitation for more than 30 years.^5^ QoL is a multi‐factor concept that encompasses subjective perceptions in the physical, psychological, social, and environmental domains.^6^ There are different tools for measuring quality of life, but a very popular one has been the Short Form‐36 (SF‐36) instrument.^7^ It is popular not only because of its complete integration of clinical features, but also because it has good psychometric properties, including high internal consistency and good test–retest reliability with people of different ages and in different states of health.^8^, ^9^ Studies have shown that stroke affects different SF‐36 domains differently,^10^, ^11^, ^12^ but their clustering has been little‐studied with stroke survivors. This study was designed to define such clustering in stroke survivors based on 8 generic SF‐36 domains.

These domains used were physical functioning (PF, 10 items), role limitation in physical activity (RP, 4 items), bodily pain (BP, 2 items), general health (GH, 5 items), vitality (VT, 4 items), social functioning (SF, 2 items), role limitation in emotional interactions (RE, 3 items), and mental health (MH, 5 items).^13^

Non‐negative matrix factorization (NMF) is a technique which has the great advantage of reducing a large number of variables into more easily interpreted latent factors shared across a large dataset.^14^ NMF was first applied in facial recognition,^15^, ^16^ then subsequently in medicine for, for example, analyzing PET images;^17^ cardiological diagnosis and risk assessment;^18^, ^19^ finding clusters of cancer subtypes;^20^ and so on. It has not so far been used for an integrated analysis of QoL subtypes. Therefore, the aim of this study was to use NMF to identify the distinct configuration of heterogeneity in stroke survivor populations on the basis of SF‐36 domains clustering. Principal component analysis (PCA) was then performed to visualize the differentiation among the subtypes, and radar charts describing the different levels of the eight SF‐36 dimensions in the subtypes were plotted.

Demographic, community, and rehabilitation‐associated factors are well understood to be important to stroke survivors' QoL. Therefore, after defining the key subtypes features, machine learning‐based feature selection using Boruta algorithms was performed to highlight the most important variables potentially linked to QoL subtypes. This combined NMF and Boruta approach helped to develop a comprehensive understanding of the demographic and environmental factors most influential in impairing QoL after a stroke. That can help to suggest therapeutic interventions including rehabilitation and social assists. To establish a prediction model, a gradient boosting machine (GBM), one of the most popular and powerful machine learning models in the industry, was applied to the data. Such machine learning techniques yield “black box” models that cannot be understood by examining their parameters.^21^ In this study, local interpretable model‐agnostic explanation (LIME) algorithms were invoked to interpret the GBM model variables that drove the predictions of QoL types. Further exploration based on LIME methods could make the model explainable and be more persuasive and dependable.^22^

**Figure 1:**
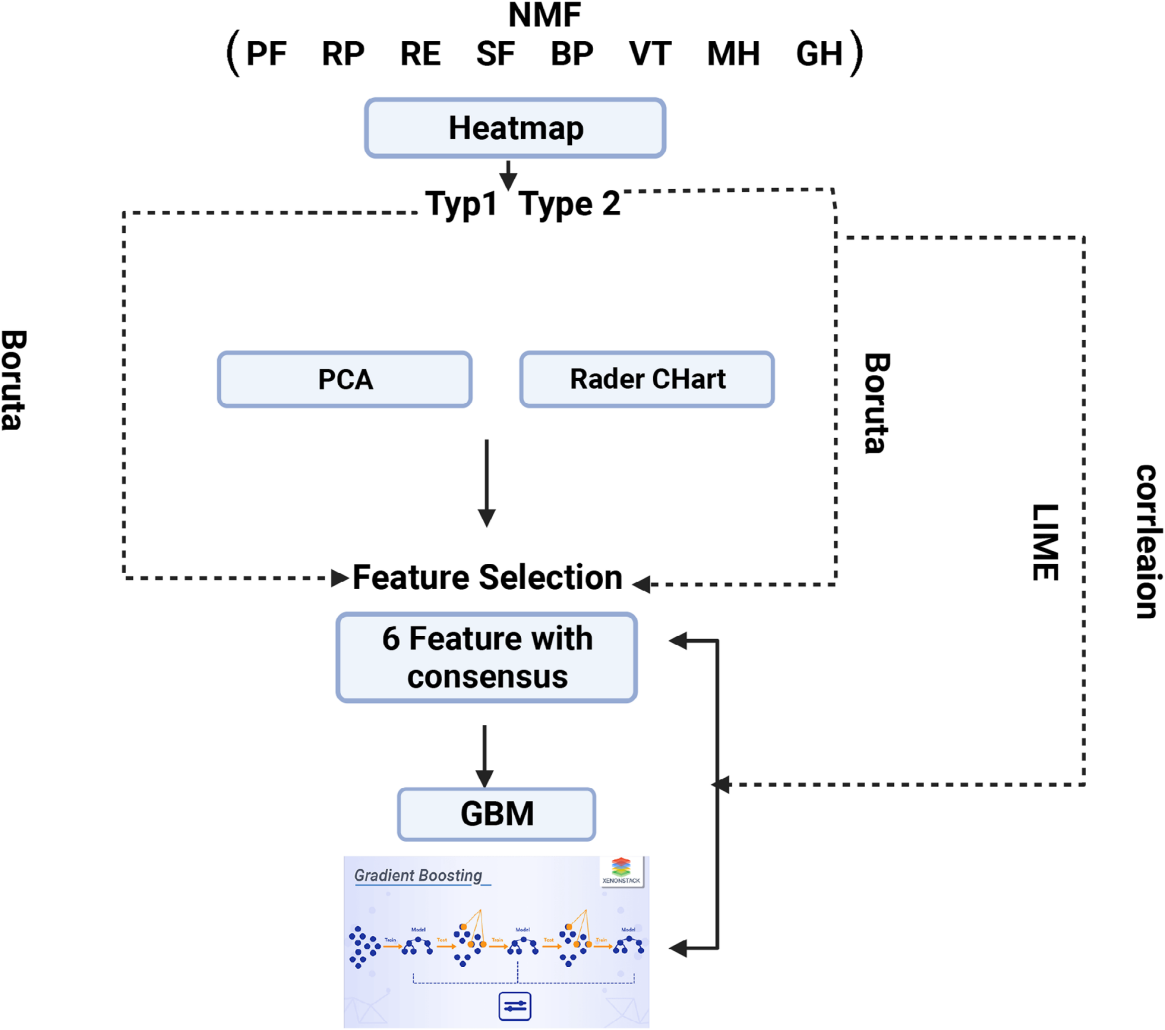
Flowchart of the study. PF, physical functioning; RP, role limitation in physical activity; BP, bodily pain; GH, general health; VT, vitality; SF, social functioning; RE, role limitation in emotional interactions; MH, mental health; NMF, non‐negative matrix factorization; PCA, principal component analysis; GBM, gradient boosting machine; LIME, local interpretable model‐agnostic explanation.

Figure 1 provides an overview of the study. Its machine learning workflow can accurately explore stroke survivor's quality of life typologies, and the factors associated with these features. This helps to understand the heterogeneity of features of impaired quality of life, as well as the pathology of the impairment.

## Methods

### Participants

The subjects in this study were stroke survivors living in the rural Xiang'an villages and towns of Xiamen City in Southern China. All had a physical disability but normal intelligence (an Abbreviated Mental Test score ≥6).^23^ The study exploited data from a cross‐sectional survey conducted from May to December 2019. Detailed information on subjects' enrollment, inclusion and exclusion criteria, and their demographic characteristics has been provided in a previous publication.^24^

### Assessment instrument scaling

Each item of the SF‐36 was scored on patient responses in the survey about their subjective impressions of their health‐related quality of life. Each domain (subscale) score was calculated using the standard SF‐36 algorithm.^25^

To assess “help from others,” the interviewers asked each subject, “are there assigned service personnel to help you in your community?” For “rehabilitation needs,” the interviewers asked whether the respondents felt that they need rehabilitation. “Opportunities for community activity” was assessed by asking whether they expected to have a chance to participate. “Community activity willingness” was assessed by asking, “are you willing to engage in community physical activity?” “Routine rehabilitation exercise” was assessed by asking each subject whether they regularly engaged in physical exercise for rehabilitation purposes.

Along with the survey, disability was graded using China's national disability classification and severity grading standards. Questions about duration since stroke and age elicited numeric responses.

### Statistical analyses

Brunet‐NMF method was used to identify potential subtypes of the physically disabled stroke survivors based on the SF‐36 domains. PCA was then applied to visualize the differences among the subtypes. The Boruta algorithm then selected out the important factors influencing the categorization of the subtypes with 1000 times iteration. The GBM's model was developed based on the Boruta selection, and we optimized the GBM's model by tuning parameters as follows: number of trees as 50, interaction depth as 2, shrinkage as 0.1, number of minobsinnode as 10. Then, the LIME method was used to interpret GBM by important feature criteria selected by “Least absolute shrinkage and selection operator (LASSO) path.” These analyses were performed with the help of version 3.6.3 of the R software suite (https://cran.r‐project.org/). The software's “mice” package was used for handling missing data.

## Results

### The SF‐36 subtypes

**Figure 2:**
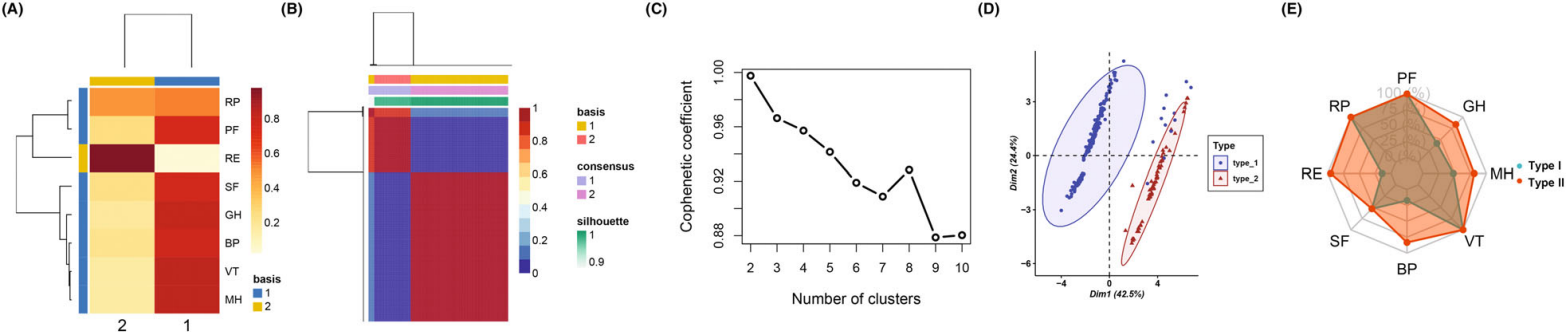
Identification of subtypes based on eight SF‐36 domains. (A) NMF basis map representing hierarchical clustering relationship among the dimensions. (B) NMF consensus map identifying 2 subtypes colored in red (silhouette coefficient >0.9). (C) NMF consensus elbow chart identifying 2 as the appropriate number of subtypes. (D) PCA plot highlighting the significant difference between the two subtypes (variance contribution of principal component 1 = 42.5%, of principal component 2 = 24.4%). (E) radar chart featuring type I versus type II, with significantly lower scores in the RE, BP, and GH domains for type I (Wilcoxon test, *p* ≤ 0.05). SF‐36, Short Form‐36 instrument; NMF, non‐negative matrix factorization; PCA, principal component analysis.

Two distinct SF‐36 subtypes were identified by non‐negative matrix factorization based on the eight‐dimensional SF‐36 data (Fig. 2), creating the basis map shown in Figure 2A and the consensus map of Figure 2B. The steep slope in the NMF consensus elbow chart (Fig. 2C) starting at 2 indicated that 2 was the appropriate number of subtype categories. On that basis, type I accounted for 74% (228) of the examples and type II accounted for 26% (80). The 2 subtypes were significantly different in visualized PCA analysis with good classification recognition (silhouette coefficient >0.9) (Fig. 2D). Type I was characterized by significantly lower role‐emotion (RE), body pain (BP), and general health (GH) scores compared with type II, as shown in the radar chart of Figure 2E. As a higher score of SF‐36 domains indicated better functioning or status, we labeled type I as worsen QoL subtype, type II as better QoL subtype.

### Predicting SF‐36 subtypes

**Figure 3:**
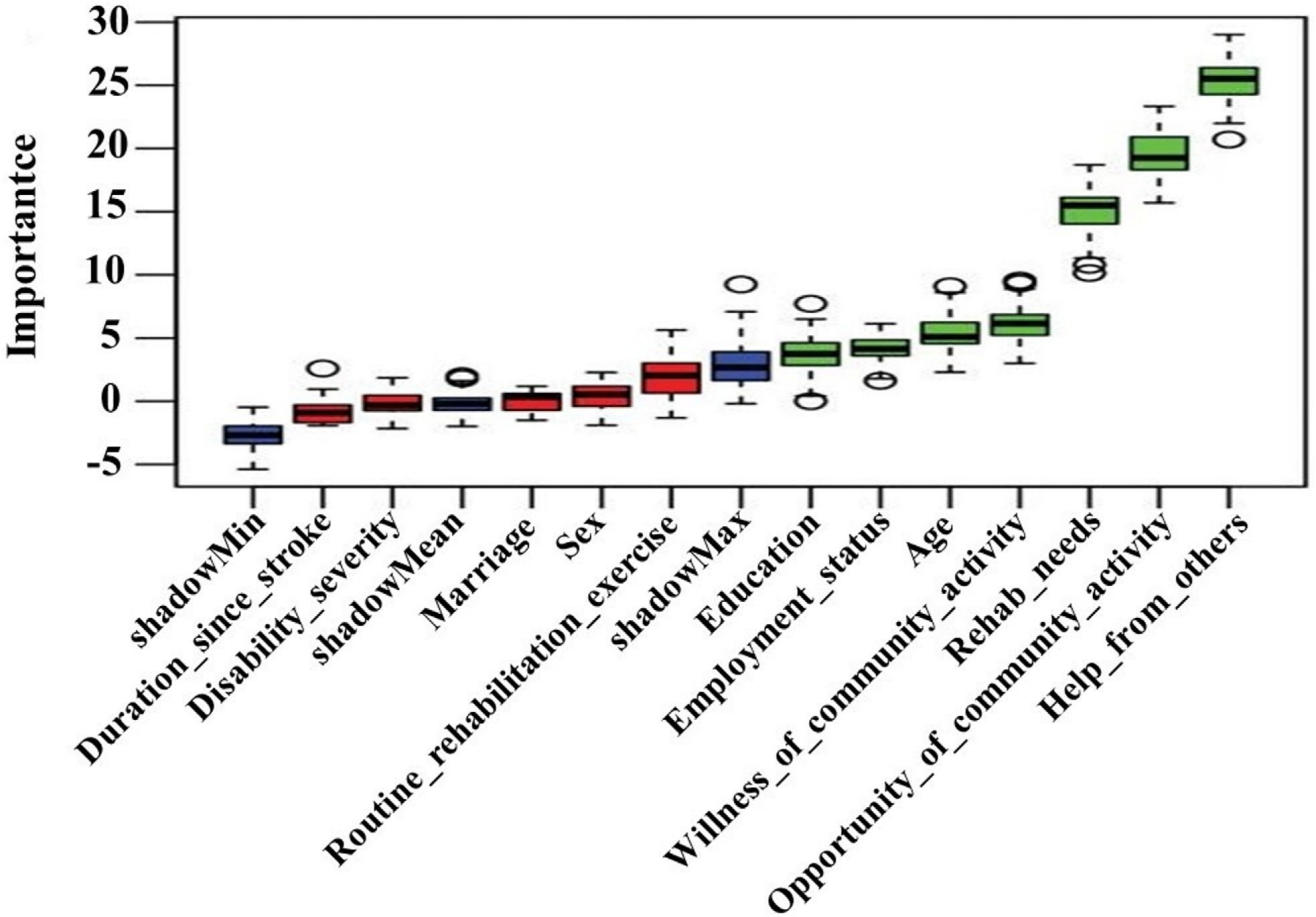
Demographic, rehabilitation‐related, and community‐related variables relevant to the classification of stroke survivors. The 7 green boxplots beyond shadowMax indicate a significant association. The importance is represented by *Z*‐values.

**Figure 4:**
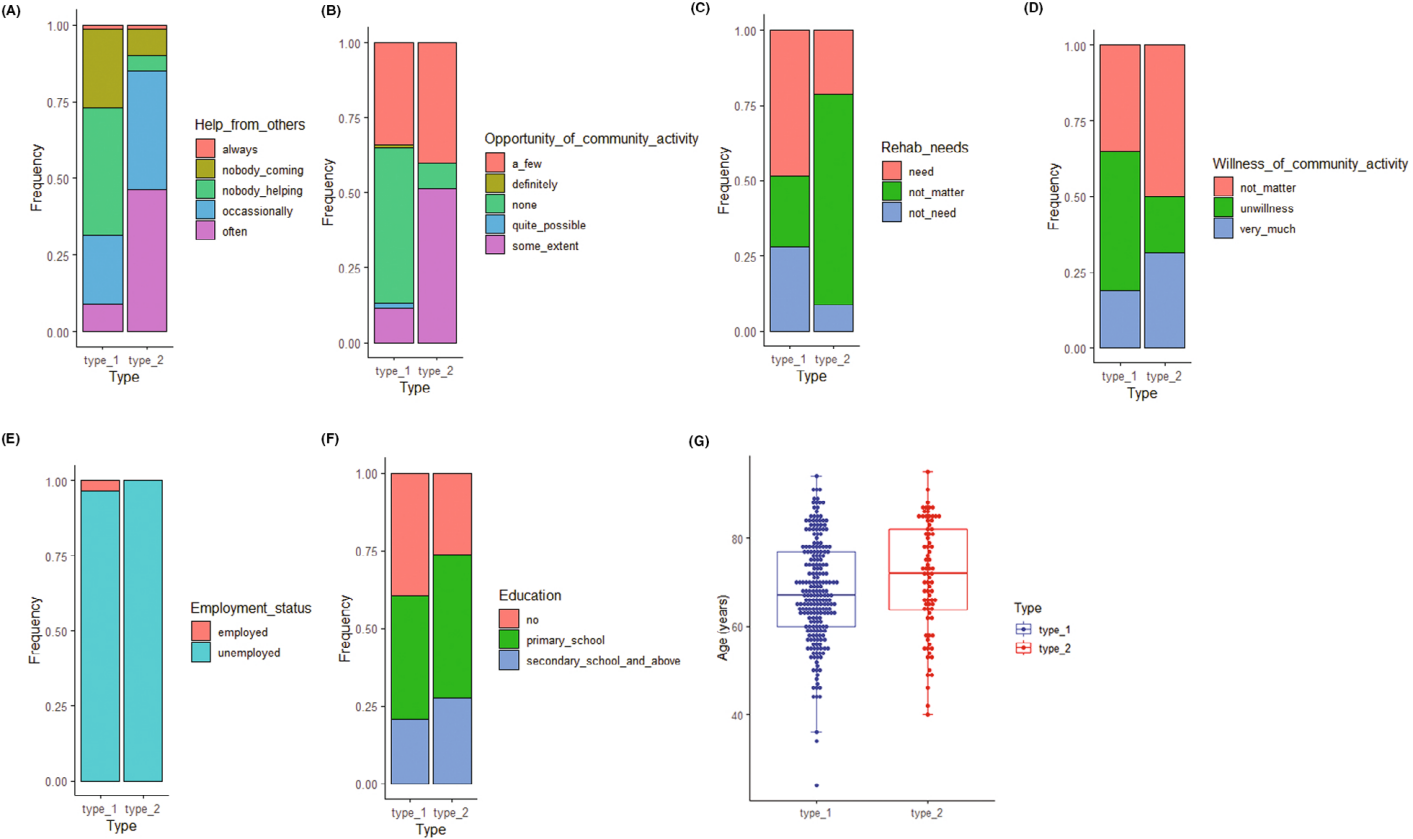
Percentage stacked bars (A–F) and a box plot (G) relating the 7 selected features with the two subtypes.

The Boruta algorithm was used to identify the demographic and community‐related variables most relevant to the classification of subtypes. Seven descriptors were identified as significantly related (Fig. 3). Help from others was the most influential, followed by opportunities of community activity, rehabilitation needs, willingness to participate in community activities, age, employment status, and education. No significant association was observed for routine rehabilitation exercise, sex, marriage, disability severity, or duration since stroke. Fig. 4 showed percentage stacked bars (Fig. 4A–F) and a box plot (Fig. 4G) relating the seven selected features with the two subtypes.

**Figure 5:**
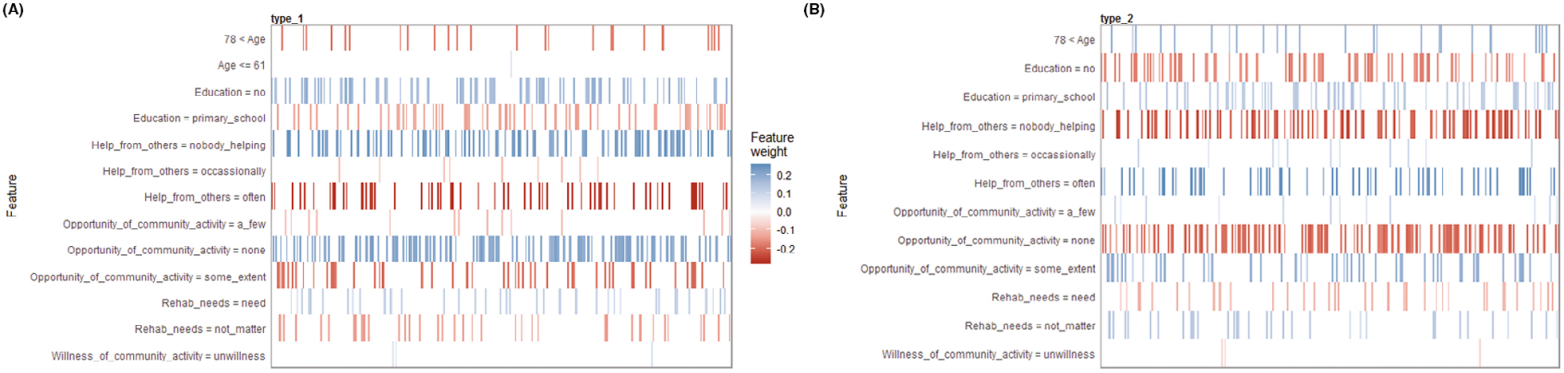
LIME graphs of the GBM model for type I (A) and for type II (B). A blue bar indicates positive influence, and a higher density blue bar indicates a greater probability of influence. The red bars indicate less likely influence, with higher density indicating a lower probability. For type I‐worsen QoL subtype, help from others (nobody helping) was the most important variable, followed by opportunities for community activities (none), education (no experience), and rehabilitation needs (need). For type II‐better QoL subtype, help from others (often) was the most important variable, followed by opportunities for community activities (some), education (primary school), and rehabilitation needs (none recognized), and age (>78 years). GBM, Gradient Boost Machine; LIME, local interpretable model‐agnostic explanation; SF‐36, Short Form‐36 instrument.

A GBM was used to model the seven selected features correlated with types I and II, and the LIME algorithm (Fig. 5) was used to interpret the GBM's model. The LIME explained how the important variables predicted (contributed to) the classifications. The sequentially‐important variables that predicted type I (worsen QoL subtype) were as follows: subjects who received little help from others, had no opportunities for community activity, limited education, and had rehabilitation needs. The factors that predicted type II (better QoL subtype) were as follows: those who received frequent help from others, had some opportunities for community activity, had a primary school education, did not feel a need for rehabilitation, and were aged over 78 years.

## Discussion

### Features of the subtypes

This study has been the first to analyze the feature subtypes based on the SF‐36 domains by NMF. It has shown that there were no significant differences between the subtypes of physically disabled stroke survivors in terms of physical function (PF), role‐physical (RP), vitality (VT), and other domains. However role‐emotion (RE), bodily pain (BP), and general health (GH) scores can significantly distinguish type I stroke survivors (as shown in Fig. 2E). This may be because some domains such as physical functioning improve to a similar degree in the overall population of stroke survivors while others do not improve uniformly. Previous studies have also shown that the physical functioning of many maybe improved after a stroke. Chen's group measured the SF‐36 results of stroke survivors immediately and after 4 weeks of treatment and reported improvement in physical variables, but still poor psychological results.^26^ Jones' team observed that only physical functioning improved in terms of SF‐36 readings during a 2‐month follow‐up.^27^

This study has for the first time demonstrated the clustering of low RE, low BP, and low GH in type I with worsen QoL stroke survivors. Previous studies have, though, similarly reported some features of this clustering: 32.9% of stroke survivors with normal cognition and language report bodily pain, most of which (81.8%) is neuropathic. In addition, the greater the neuropathic pain, the greater the deterioration in mental quality of life.^28^ The SF‐36 results were more severely impaired in stroke survivors with depression, especially in the role‐emotion (RE) and social functioning (SF) domains.^29^ It is worth mentioning that in this study the mental health (MH) and SF readings of the type I patients were slightly lower on average than those of the type II patients. The difference was not statistically significant, but it might prove to be so with a larger sample in future research.

Early screening and appropriate management of emotional difficulties are key to preventing long‐term complications in stroke survivors.^30^ However, emotion does not equal RE, which refers to role limitations in emotional interactions, meaning a person's belief in their ability to accomplish something. RE belongs to self‐perceived behavior control,^31^, ^32^ which is the key factor affecting the behavior change based on planned behavior theory.^33^ Study has shown that self‐control can promote functional recovery in stroke survivors, while improving mood can enhance physical abilities.^34^ Therefore, establishing the effectiveness of emotions in behavioral control (healthy RE) is important for improving function and subsequent quality of life, although RE has not received much attention in secondary prevention of stroke.

Effective pain interventions also need to be developed for stroke survivors. Survey finds that majority of the stroke survivors (90.1% total 322) received treatment for pain; however, it is often ineffective: among them, 79.8% received drug treatment, and the effective rate was 47.1%; 64.6% received nondrug treatment with an effective rate of 52.4%; nondrug treatment includes physiotherapy was the most common therapy (48.1%), followed by occupational therapy (15.5%) and psychology (11.8%).^35^ Our study found that body pain was one of the characteristics of type I, and that the related factors (underlying pathology) of type I were insufficient helping, inadequate community opportunities, and unmet rehabilitation needs, etc. Therefore, targeting these related factors may be effective in alleviating type I symptoms, including body pain. This confirms the importance of nondrug treatments for pain. Understanding stroke survivors typology will promote more effective and integrated rehabilitation.

### Community and demographic factors predicting quality of life

This study has found that “help from others” and “opportunities for community activities” are the most important predictors of quality of life. Previous studies have shown that social support and community involvement are interrelated, and either is strongly associated with quality of life and also depression.^36^ Depression in a stroke survivor is likely to be reflected in a low role‐emotion (RE) score, which is characteristic of type I individuals with a poor quality of life. Previous studies have shown that social support is a significant predictor of quality of life, mediated by poststroke depression.^36^ Among aphasic stroke survivors, depression is known to be strongly correlated with decreased community participation and impaired quality of life.^37^ Social support is an important predictor of successful community participation.^38^, ^39^ The results reported here thus make sense: “little help from others” and “no opportunities for community involvement” are predictors of type I (worsen QoL subtype); “often getting help from others” and “some opportunity for community involvement” are predictors of type II (better QoL subtype).

It can be expected that type I participants with poor quality of life may experience substantial challenges in overcoming body pain (BP), role‐emotion (RE), and worse overall sense of general health (GH) as they were likely to have gain opportunity of community activities. Therefore, policy and infrastructure changes to promote social support and community engagement for people with low BP, RE, and GH are a top priority to increase their community opportunities and reduce health disparities among stroke survivors with disabilities.

Next key predictor for the quality of life subtypes is “rehabilitation need.” Studies have shown that rehabilitation such as task‐based exercises, strength and endurance training, and aerobic exercise can benefit the reorganization of brain function and improve the energy levels of stroke survivors. Both inpatient and community rehabilitation are therefore important.^40^ However, assessing the complex global rehabilitation needs after a stroke is a challenge.^41^ This study found that “need for rehabilitation” was associated with type I with worsen QoL (predictably), but not feeling in need of further rehabilitation was associated with type II with better QoL. Specifically, 228 of the 308 physically disabled stroke survivors studied (74%), did subjectively consider further rehabilitation to be necessary. Studies have shown, similarity, that rehabilitation has benefits for the emotions, for pain, and for overall health: exercise therapy and electrotherapy are effective in dealing with complex local pain syndromes after a stroke;^42^ aerobic treadmill training or walking outdoors can relieve depression and improve endurance, mobility, and quality of life in general.^43^ Therefore, the characteristics of type I (low RE, BP, GH) could help identify people in need of rehabilitation.

The literature shows that people with disabilities who have the greatest needs are also those whose health care systems are least likely to meet those needs.^44^ Similarly reported in our study, the social and rehabilitation needs of type I with worsen QoL were overlooked or delayed, possibly due to a lack of heterogeneous descriptions of clinical quality of life manifestations, also may be because impaired MH, RE, and GH could often be obscured by clinical aspects of bodily functions that people normally value. Once they do not have access to timely treatment and standardized secondary prevention of stroke impaired QoL, they could have a worse prognosis than those who receive correct intervention. Therefore, identifying type I individuals with poor quality of life is critical and policy interventions are urgently needed to improve their quality of life.

Lack of education was also a predictor of type I with worsen QoL classification, while at least a primary education and being older than 78 were predictors of type II with better QoL. This can be explained by the fact that the better‐educated would tend to have higher RE scores,^24^ probably because education fosters flexible coping skills that make survivors more adaptable to challenges.^45^ In addition, research has shown that older persons tend to have higher RE scores.^24^ It may be that older stroke survivors have lower physical health expectations and may thus cope better emotionally.^46^ Physical functioning is normally impaired in older adults, but type II is based on good RE, BP, and GH scores, rather than on physical functioning. The important role of RE in the types of stroke survivors encourages us to pay attention to education, knowledge and skill development and other behavioral aspects in rehabilitation management to improve the quality of life, especially for the type I stroke survivors.

Interestingly, the results show that neither the duration since stroke nor disability severity was a subtype predictor. This means that type I characteristics (low RE, BP, and GH) may be maintained or changed at any stage, regardless of the course of the disease or the severity of the disability. Research has shown that some stroke survivors maintain the same level of quality of life during at least 2.5 years after discharge, with the ratio of improvement to deterioration remaining roughly the same on the “anxiety/depression” dimension. There are, though, twice as many deteriorating as improving on the “pain/discomfort” dimension.^47^ Another study has shown that quality of life does not decrease in tandem with stroke severity. In addition, severity is also not a significant predictor of anxiety or depression.^48^ This inspires a resolution to intervene with type I with worsen QoL individuals at any stage, regardless of the severity of the disability. This study's clustering should be stable regardless of the duration of the disease and the degree of disability. Therefore, the subtype analysis developed here may serve as a useful new medical evaluation tool to improve the rehabilitation effect and quality of life of stroke survivors.

### Limitations

It is of course important to recognize that the population studied all lived in rural southern China. Multi‐region or even international data would be better to generalization of the findings. In addition, this was a cross‐sectional study which could not prove causality. And finally, the outcome of the measurement was reported subjectively, although interviewers and data collectors were well trained, results bias still could remain.

### Conclusions

NMF was used to cluster feature types among 308 physically disabled stroke survivors in rural China. There were 2 distinct subtypes of quality of life, with 74% of respondents classified as type I with worsen role‐emotion (RE), physical pain (BP), and general health (GH) scores on the SF‐26 instrument. The other domains such as physical function (PF) did not distinguish between the subtypes. RE, BP, and GH should therefore be targeted for intervention regardless of the state of a stroke survivor's physical functioning.

The model developed using a GBM with Boruta selection and the LIME algorithm explained that the most important variables driving type classification should be help from others, opportunities to participate in community activities and need for rehabilitation (all self‐perceived). Disease duration and disability severity are not relevant to subtype classification. Social support, community engagement, and rehabilitation services are what can best improve the quality of life of stroke survivors of type I, no matter the course of the disease and disability severity.

Machine learning has been shown to accurately cluster features and make predictions given sufficiently voluminous data. Identifying subtype characteristics and their predictors for quality of life will help professionals and society develop more precise prevention and rehabilitation strategies.

Clinical implications suggest that the significance of long‐term recovery in stroke survivors with physical disabilities extends beyond motor deficits. This notion is crucial as it emphasizes the impact of physical pain and emotional‐behavioral issues on patients' ability to attain adequate rehabilitation needs and reintegrate into the community. Interventions should prioritize addressing the unmet medical and community needs of these individuals, facilitating the development of their sense of belonging and identity. It is important to note that poststroke chronic pain and emotional‐behavioral problems can be severe and pose challenges in terms of treatment.

## Author Contributions

All authors have participated in the research project conception, design, review & editing the manuscript. QX contributed to the conception, design, manuscript drafting, and methodology; LL contributed to the investigation, project administration, and supervision; TY contributed to the resources, supervision, and editing the manuscript; JH contributed to the funding acquisition, supervision, and project administration; ZL contributed to the conception, formal analysis and methodology; WZ contributed to the formal analysis and methodology; XW and DZ contributed to the investigation, data curation and validation; TL contributed to the investigation and funding acquisition. All authors have read and agreed to the published version of the manuscript.

## Conflict of Interest

The authors declare no conflicts of interest.

## Data Availability

All of the data and details of the analyses can be requested from the corresponding author (T.Y.).
